# ngs.plot: Quick mining and visualization of next-generation sequencing data by integrating genomic databases

**DOI:** 10.1186/1471-2164-15-284

**Published:** 2014-04-15

**Authors:** Li Shen, Ningyi Shao, Xiaochuan Liu, Eric Nestler

**Affiliations:** 1 Fishberg Department of Neuroscience and Friedman Brain Institute, Icahn School of Medicine at Mount Sinai, New York, New York 10029, USA

**Keywords:** Next-generation sequencing, Visualization, Epigenomics, Data mining, Genomic databases

## Abstract

**Background:**

Understanding the relationship between the millions of functional DNA elements and their protein regulators, and how they work in conjunction to manifest diverse phenotypes, is key to advancing our understanding of the mammalian genome. Next-generation sequencing technology is now used widely to probe these protein-DNA interactions and to profile gene expression at a genome-wide scale. As the cost of DNA sequencing continues to fall, the interpretation of the ever increasing amount of data generated represents a considerable challenge.

**Results:**

We have developed ngs.plot – a standalone program to visualize enrichment patterns of DNA-interacting proteins at functionally important regions based on next-generation sequencing data. We demonstrate that ngs.plot is not only efficient but also scalable. We use a few examples to demonstrate that ngs.plot is easy to use and yet very powerful to generate figures that are publication ready.

**Conclusions:**

We conclude that ngs.plot is a useful tool to help fill the gap between massive datasets and genomic information in this era of big sequencing data.

## Background

Next generation sequencing (NGS) technology has become the *de facto* indispensable tool to study genomics and epigenomics in recent years. Its ability to produce more than one billion sequencing reads within the timeframe of a few days [1] has enabled the investigation of tens of thousands of biological events in parallel [2,3]. Applications of this technology include ChIP-seq to identify sites of transcription factor binding and histone modifications, RNA-seq to profile gene expression levels, and Methyl-seq to map sites of different types of DNA methylation with high spatial resolution, among many others. To convert these data into useful information, the sequencing reads must be aligned to reference genomes so that coverage – the number of aligned reads at each base pair – can be calculated. A genome browser is a very handy tool that can be used to visualize the coverage along with other genomic annotations, such as genes, repeats, conservation scores, and genetic variants as stacked tracks [4,5]. Designing a genome browser that can effectively manage the enormous amount of genomic information has become an important research topic in the past decade with dozens of tools being developed to date [6-8].

As more NGS data are being generated at reduced cost [9], researchers are starting to ask more detailed questions about these data. For example, after ChIP-seq data for a given histone modification (“mark”) is generated, one might ask: 1. What is the enrichment of this mark at transcriptional start sites (TSSs) as well as several Kb up- and down-stream? 2. If a ranked gene list is obtained based on the enrichment of this mark, does it associate with gene expression? 3. Does this mark show any co-occurrence with other marks and do their co-enrichments define gene modules? To answer these and many additional questions, it would be very helpful to retrieve the coverage for a group of functional elements together, perform data mining on them, and then visualize the results. Classic examples of functional elements include TSSs, transcriptional end sites (TESs), exons, and CpG islands (CGIs). With the availability of high-throughput assays, novel functional elements – such as enhancers and DNase I hypersensitive sites (DHSs), are being discovered by computational programs at a very rapid pace. Progress is being facilitated further by the human ENCODE project [10,11], where researchers found recently that ~80% of the human genome is linked to biochemical functions.

On the other hand, the development of tools that can be used to explore the relationships between NGS data and functional elements within the genome has lagged. Some programs [12,13] have incorporated simple functions for a user to generate average profile plots at TSSs, TESs, or genebody regions, but with very limited options to customize the figures. A few program libraries [14-17] have been developed to facilitate the calculation and plotting of coverage from NGS data, but they require a user to have substantial programming skills and involve a steep learning curve. Several programs [18-20] with graphical interfaces have been developed, featuring a point-and-click workflow to perform these tasks. They are greatly helpful for investigators with limited programming experience. However, their designs often limit the choices a user has and it is not always easy to import and export data from these programs.

To address this important need, we have developed ngs.plot: a quick mining and visualization tool for NGS data. We tackle the challange in two steps. Step one involves defining a region of interest. We have collected a large number of functional elements from major public databases and organized them in a way so that they can be retrieved efficiently. The ngs.plot database now contains an impressive number of 60,520,599 functional elements (Table 1). Step two involves plotting something meaningful at this region. Our program utilizes the rich plotting functionality of R [21] and contains 27 visual options for a user to customize a figure for publication purposes. ngs.plot’s unique design of configuration files allows a user to combine any collection of NGS samples and regions into one figure.

**Table 1:** Summary statistics of the ngs.plot database

**Item**	**Count**	**Description**
Annotation sources	4	Refseq, Ensembl, ENCODE, muENCODE
Species (Genome)	17(21)	Human (hg18, hg19), chimpanzee (panTro4), macaque (rheMac2), mouse (mm9, mm10), rat (rn4, rn5), cow (bosTau6), horse (equCab2), chicken (galGal4), zebrafish (Zv9), drosophila (dm3), *Caenorhabditis elegans* (ce6, ceX), *Saccharomycer cerevisiae* (sacCer3), *Schizosaccharomyces pombe* (Asm294), *Helicobacter pylori* (Asm852v1), *Sulfolobus acidocaldarius* (sulfAcid1), *Arabidopsis thaliana* (TAIR10), Zea mays (AGPv2)
Biotypes	7	TSS, TES, genebody, exon, CGI, DHS, enhancer
Gene type	5	Protein coding, lincRNA, miRNA, pseudogene, misc (everything else)
Exon types	7	canonical, promoter, polyA, variant, altDonor, altAcceptor, altBoth
CGIs	10	Hg18, hg19, mm9, mm10, rn4, rn5, bosTau6, galGal4, panTro4, rheMac2
Enhancers	9 (hg19)	Url: http://hgdownload.cse.ucsc.edu/goldenPath/hg19/encodeDCC/wgEncodeBroadHmm/
		Cell types: H1hesc (default), Gm12878, Hepg2, Hmec, Hsmm, Huvec, K562, Nhek, Nhlf.
	15 (mm9)	Url: http://chromosome.sdsc.edu/mouse/download.html. Cell types: mESC, bone marrow, cerebellum, cortex, heart, intestine, kidney, liver, lung, MEF, olfactory bulb, placenta, spleen, testes, thymus.
DHS	125 (hg19)	Url: http://hgdownload.cse.ucsc.edu/goldenPath/hg19/encodeDCC/wgEncodeAwgDnaseUniform/. Cell types: H1hesc, A549, Gm12878, Helas3, Hepg2, Hmec, Hsmm, Hsmmtube, Huvec, K562, Lncap, Mcf7, Nhek, Th1,
Region analysis	8	ProximalPromoter, Promoter1K, Promoter3K, Genebody, Genedesert, OtherIntergenic, Pericentromere, Subtelomere

Total count of functional elements is 60,520,599.

The ngs.plot package contains multiple components: a main program for region selecting and plotting; a genome crawler that grabs genomic annotations from public databases and packs them into archive files; a script that is used to manipulate the locally installed genomic annotation files; another script that can be used to calculate and visually inspect correlations among samples; a plug-in that allows ngs.plot to be integrated into the popular web-based bioinformatic platform – Galaxy [22]. ngs.plot has been developed as an open-source project and has already enjoyed hundreds of downloads world-wide thus far. Here, we will first describe the design and implementation of ngs.plot. We will then discuss some implementation strategies by using performance benchmarks. Finally, we will employ a few examples to demonstrate how ngs.plot can be used to extract and visualize information easily, with rich functionality in plotting.

## Implementation

### ngs.plot workflow and algorithms

**Figure 1:**
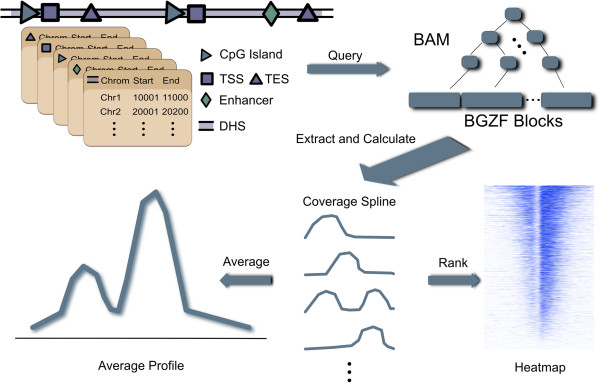
**The workflow of an ngs.plot run.** The functional elements in the database are classified based on their types, such as TSS, CGI, enhancer, DHS. The genomic coordinates of the functional elements are used to query a BAM file which is indexed by an R-tree like data structure. Coverage vectors are calculated based on the retrieved alignments, which are further represented as average profiles or heatmaps.

The workflow of ngs.plot is depicted in Figure 1. Initially, ngs.plot searches through its database to find the genomic coordinates for the desired regions and uses them to query the alignment files of an NGS dataset. It then calculates the coverage vectors for each query region based on the retrieved alignments. It finally performs normalization and transformation on the coverage and generates two plots. One plot is an average profile that is generated from the mean of all regions. This plot provides the overall pattern at the regions of interest. The other plot is a heatmap that shows the enrichment of each region across the genome using color gradients. The heatmap can provide three-dimensional details (enrichment, region, and position) of the NGS samples under study.

A user can specify the plotting regions using a genome name, such as “mm9” and a region name, such as “genebody”. Further options are provided to choose a particular type of region, otherwise the default is used. For example, exons are classified into “canonical” (default), “variant”, “promoter”, etc.; CGIs are classified into “ProximalPromoter” (default), “Promoter1k”, “Promoter3k”, etc.; gene lists can be provided to create subsets of the regions. For convenience, we have provided the gene names/IDs in both RefSeq [23] and Ensembl [24] format. To be more flexible, a user can also use a BED (https://genome.ucsc.edu/FAQ/FAQformat.html#format1) file for custom regions. A BED file is a simple TAB-delimited text file that is often used to describe genomic regions. This is particularly useful if a user performed peak calling for a transcription factor and would like to know what is happening at or around the peaks.

The alignment files must be in BAM [25] format, which is now used widely for short read alignments. ngs.plot conforms to the SAM specification [25] of BAM files and can work with any short read aligner. A BAM file is compressed and indexed for efficient retrieval. In ngs.plot, the “physical coverage” instead of the “read coverage” is calculated for both ChIP-seq and RNA-seq. This is achieved by extending each alignment to the expected DNA fragment length according to user input. The coverage data are then subjected to two steps of normalization. In the first step, the coverage vectors are normalized to be equal length and this can be achieved through two algorithms. The default algorithm is spline fit where a cubic spline is fit through all data points and values are taken at equal intervals. The alternative algorithm is binning where the coverage vector is separated into equal intervals and the average value for each interval is calculated. This first step of length normalization allows regions of variable sizes to be equalized and is particularly useful for genebody, CGI, and custom regions. In the second step, the vectors are normalized against the corresponding library size – i.e., the total read count (only the reads that pass quality filters are counted) for an NGS sample to generate the so called Reads Per Million mapped reads (RPM) values. The RPM values allow two NGS samples to be compared regardless of differences in sequencing depth.

We have implemented many functions to manipulate the visual outputs of an ngs.plot run, as follows:

#### RNA-seq mode

**Figure 2:**
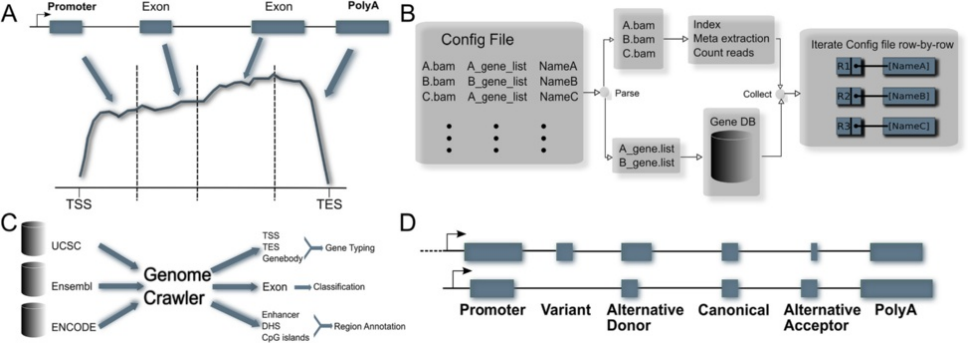
**Design and implementation of the ngs.plot program. A**. In RNA-seq mode, the program performs exon splicing *in silico*: the coverage vectors for exons are concatenated with intronic coverage removed. **B**. A configuration file can be used to create any combination of BAM files and regions. The program will parse the configuration and perform pre-processing on BAM files. It will then iterate through each line of the configuration and determine the arrangement of the output figure. **C**. A genome crawler is developed to automatically pull genomic annotations from three public databases – UCSC genome browser, Ensembl and ENCODE. It then performs more elaborate classifications on the functional elements and compiles them into R binary tables. **D**. The exon classification algorithm classifies exons into seven categories: promoter, variant, alternative donor, alternative acceptor, alternative both, and polyA based on pairwise comparisons of exon boundaries.

ngs.plot can accurately calculate coverage for RNA-seq (Figure 2A). RNA-seq experiments are unique because the short reads are derived from messenger RNAs and other expressed RNAs, many of which result from exon splicing. The ngs.plot database contains the exon coordinates for each transcript so that the coverage vectors for exons are concatenated to simulate RNA splicing *in silico*.

#### Bam-pair

ngs.plot can also calculate the log2 ratios for one sample vs. another and display the values using two different colors in a heatmap. This is a very useful feature for ChIP-seq where a target sample is often contrasted with a control sample to determine *bona fide* differences in enrichment.

#### Visualization options

We have implemented a few approaches to generate average profiles. Besides mean values, the standard error of mean (SEM) across the regions is calculated and shown as a semi-transparent shade around the mean curve. This provides users with a sense of statistical significance when two samples are being compared. It is known that the mean value is most influenced by extreme values that can sometimes deleteriously distort the average profiles. We therefore implemented robust statistics (as an optional feature) by removing a certain percentage of the extreme values before the average is taken. As well, curve smoothing was implemented to remove the spikes from average profiles as an option that can be controlled by moving window size. Heatmaps can be tuned by custom color scales and color saturation.

#### Gene ranking

In contrast to an average profile, a heatmap contains an additional dimension – individual genomic regions. This additional information allows the regions to be organized to reflect the underlying biology. We have therefore implemented six different algorithms to rank such regions:

• Total (default). Regions are ranked by the sum of the enrichment values. This always puts the most enriched regions at the top.

• Hierarchical clustering. This method groups the most similar regions together first followed by the less similar ones. This process is performed repeatedly from bottom up until all regions are included in the grouping to form a tree-like structure. When dealing with multiple NGS samples, the clustering is applied to all of them together.

• Max. Regions are ranked by the maximum of the enrichment values. This is similar to the “Total” algorithm but is most useful when dealing with epigenomic marks that have sharp peaks.

• Product. Regions are ranked by the product of the sums of all NGS samples. This algorithm is useful when a user is studying several marks that may act in concert with one another.

• Difference. Regions are ranked by the difference of sums between two NGS samples. When two marks are mutually exclusive, such as H3K27ac and H3K27me3, this algorithm can maximize the appearance of such relationships.

• Principal component analysis (PCA). PCA is performed on all NGS samples and then the first component is used to rank regions, which captures the largest proportion of the variance. This algorithm is complementary to the above mentioned methods.

Finally, a user can choose not to rank the regions and just use the input order (called “none”). This is particularly useful if a user has already ranked the regions. For example, a user can rank genes by expression levels and then plot the enrichment for histone marks to see if there is any association.

#### Multi-plot and configuration

In a multi-plot, an arbitrary number of plots can be combined into one figure and each plot can represent an NGS sample at a subset of the entire genomic region; a configuration file can be used to describe this combination. The configuration is a TAB-delimited text file where the first column contains the alignment file names; the second column contains the gene list names or BED file names; the third column contains the titles of the plots; the fourth and fifth columns are optional and contain fragment lengths and custom average profile colors, respectively. ngs.plot will parse a configuration file and obtain a list of unique BAM files and a list of unique regions (Figure 2B). Some pre-processing steps will be performed on each BAM file, such as calculating the number of alignments and indexing. The unique regions and unique BAM files are used to organize heatmaps into a grid so that each row represents a unique region and each column represents a BAM file.

#### Other tools

Included in the ngs.plot package are several additional useful tools. A Python script called ngsplotdb.py can be used to install downloaded genome files, list currently installed genomes, or remove existing genomes. An R script called plotCorrGram.r can be used to calculate all pairwise correlations for samples in a configuration and visually display them as a corrgram [26]. Another R script called replot.r can be used to re-generate an average profile or a heatmap with different visual options so that users can tune their figures without extracting data again.

### Coverage extraction

Coverage extraction is at the core of the ngs.plot workflow. This process often consumes a lot of computational resources because of the large size of genomes (e.g., the human genome has approximately 3 billion nucleotides) and because alignment files are also very large (on the order of tens of GB). In the history of ngs.plot, we first used a strategy called “run-length encoding” (RLE) to represent genomic coverage vectors. RLE uses a very simple approach so that consecutive and repetitive values are represented by the value and number of repeats. For example,

#### Original

000000000011111222223333300000000.

#### RLE

(0,10) (1,5) (2,5) (3,5) (0,8).

This leads to very efficient representation if the original coverage vectors are sparse. For histone marks, such as H3K4me3, which tends to generate sharp peaks, a run-length encoded 10 million short read sample only occupies ~15 MB on a hard-disk if stored as a binary file. However, as sequencing output has increased rapidly in recent years (which inevitably creates values at originally zero-value regions), this strategy soon became a major problem: the RLE files grew too large and consumed a lot of memory during loading. Another challenge arose when dealing with epigenomic marks that have broad patterns of enrichment – the coverage vectors are dense and may consume a lot of memory.

Therefore, we developed another strategy that uses a two-step procedure (Figure 1). First, the query regions are grouped into chunks and the BAM index is loaded into memory to perform alignment retrieval. Second, the retrieved alignments are used to calculate coverage on-the-fly for each region. A BAM file is indexed using hierarchical binning and linear index to allow very efficient retrieval so that only one disk seek (moving the disk head to the desired location) is often required for each query [25,27]. Grouping regions into chunks allows us to avoid frequent index loading which is very expensive in comparison to alignment reading. This strategy has an additional advantage: no extra files need to be generated to represent coverage vectors. When the storage of many NGS samples becomes problematic, this advantage is highly desirable.

We also explored additional alternatives (see Benchmarking the performance of ngs.plot section). We used samtools to pre-calculate the genomic coverage vector for an NGS sample, merged the neighbouring base pairs that contain the same value, and compressed them using gzip to save space. We then used two different approaches to index the output file. Tabix [27] is a generic indexing program for TAB-delimited text files that contain a position column and a value column, and uses the same indexing algorithm as BAM. It can directly create an index on a compressed text file. bigWig [28] files are converted from wiggle (http://genome.ucsc.edu/goldenPath/help/wiggle.html) files. It is a binary format that includes a data structure called R-tree as index. We first converted the output file to a variable-step wiggle file and then created the bigWig file using tools from the UCSC genome browser.

### Genomic annotation databases

We developed a genome crawler that fetches various genomic annotations from public databases, and processes and saves them into R binary tables (Figure 2C). R binary tables are very easy to create and their columns are indexed by R internally. This helps to avoid setting up local databases, which turns out to be a convenience for users. Currently, we considered Ensembl [24], UCSC [29], and ENCODE [11] [see Additional file 1: Table S2], and will incorporate more public databases in the future. Ensembl and UCSC provide classic genomic features such as genes, transcripts, exons, and CGIs, while ENCODE provides more recent epigenomic features such as enhancers and DHSs. Because these databases host genomic information at different servers that are setup by separate groups of people, there is no uniformity in constructing the URL for a specific genome. Sometimes, a large database (such as Ensembl) may store different classes of species, such as animal and plant, using slightly different naming schemes. To address this issue, we used JSON format to manually create configuration files for each naming scheme so that an automated pipeline can pull data from different sources. New naming schemes can be handled by simply adding JSON configuration files. The files that are downloaded by the genome crawler include Gene Transfer Format (GTF), Gene Prediction (GP), BED, and MySQL database inquiries, each of which is processed by a separate program module. The RefSeq annotations downloaded from UCSC are in GP format, which can be converted into GTF files using the “genePredToGtf” utility from UCSC. The GTF files are parsed by custom scripts to generate uniformly formatted text files that are further converted into R binary tables. The gene annotations are used to derive gene deserts. Locations about heterochromatic regions such as centromeres and telomeres are downloaded from UCSC and are used to derive pericentromeres and subtelomeres. All the gene annotations, gene deserts, pericentromeres and subtelomeres are used to build a genome package for the “region analysis” utility (https://github.com/shenlab-sinai/region_analysis) on the fly, which is used to perform location-based classifications on CGIs and DHSs. In total, more than 60 million functional elements have been incorporated into ngs.plot’s database so far (Table 1). Additional genomes can be added at any time as needed. The functional elements for each genome are packed into a compressed archive file that can be installed on demand by a user. A Python script (named ngsplotdb.py) is provided to manage the locally installed genomes. In the following, we describe each type of functional element and how they are processed.

#### Genes and transcripts

Genes and transcripts are categorized into five types: protein_coding, pseudogene, lincRNA, miRNA, and misc (everything else) according to GTF files. Gene/transcript IDs/names are indexed for random access. Each gene is represented by the isoform with the longest genomic span.

#### Exons

Exons and their neighbouring regions are known to contain chromatin modifications that may facilitate exon recognition and influence alternative splicing [30-32]. We thus developed an exon classification algorithm [see Additional file 1] that classifies each exon into seven categories (Figure 2D):

• Promoter: the 5’ end.

• PolyA: the 3’ end.

• Canonical: common to all isoforms of the gene.

• Variant: absent from some isoforms.

• Alternative donor: have varied 3’ end.

• Alternative acceptor: have varied 5’ end.

• Alternative both: have both varied 3’ and 5’ ends.

The first two categories are terminal exons while the other five categories are internal exons. Briefly, our algorithm goes through each gene and carries out pairwise comparisons for all transcripts within the gene. All exons are initialized to “canonical” category and will be continuously updated when the program sees alternative boundaries or missing exons in comparison to other transcripts.

#### Enhancers

Enhancers are important transcriptional regulators that can activate distal promoters via DNA looping. They often regulate subsets of genes in a cell type specific way and are marked in part by the enrichment of H3K4me1 and H3K27ac [33,34]. We have built into our database the enhancers of 9 human cell types and 15 mouse cell types (Table 1) by using data from the ENCODE [33] and muENCODE projects [34]. For human enhancers, we incorporated data from the ENCODE Analysis Working Group (AWG) which performs integrated analysis of all ENCODE data types based on uniform processing. We will continuously monitor the status of their download page and update our database as new data become available. We excluded the enhancers that are within ±5 Kb of TSSs. The distance of 5 Kb is a cutoff inspired by this work [33] to avoid classifying promoters as enhancers accidentally. Each enhancer is assigned to their nearest genes whose IDs/names are also indexed.

#### DHSs and CGIs

DHSs are thought to be characterized by open, accessible chromatin and are functionally related to transcriptional activity. DHSs have been used as markers of regulatory DNA regions [35,36] including promoters, enhancers, insulators, silencers, and locus control regions. High-throughput approaches, namely DNase-seq (using NGS) and DNase-chip (using tiled microarrays), were used to map DHSs on the human genome [37]. In ENCODE, DNase-seq was recently used to map genome-wide DHSs in 125 human cell and tissue types [38]. We have built into ngs.plot’s database the DHSs of 125 human cell types (Table 1) from the download page provided by AWG and will update them in the future. CGIs are genomic regions that contain high frequency of CpG sites and are often involved in gene silencing at promoters. CGIs are provided in ngs.plot (Table 1) based on the annotations from the UCSC genome browser. Both DHSs and CGIs are classified into different groups based on their genomic locations using the region analysis utility.

### Galaxy integration

ngs.plot command interface features simple and easy usage. This allows users to blend ngs.plot with other bioinformatic and Unix tools seamlessly. However, the command interface may be intimidating to wet lab biologists. Therefore, we developed a plug-in so that ngs.plot can be integrated into Galaxy [22] – a very popular web-based bioinformatics platform, which allows users to build their own point-and-click workflows using various tools. The plug-in features an easy-to-use graphic interface that can typically generate a figure in 3-4 steps. We have created a wiki-page to demonstrate such an example: https://code.google.com/p/ngsplot/wiki/webngsplot. Currently, this plug-in requires a locally installed Galaxy instance and is not available on the main Galaxy server.

### Website and community involvement

ngs.plot’s hosting website provides manuals, source code, installation files, and links to many other resources. The source code is tracked by Google’s git server and is open for public contributions. To facilitate users in using ngs.plot, we have created nine wiki-pages so far and will keep adding new ones. Issue tracking is used for users and developers to report bugs and make suggestions. As this manuscript is being written, users from all over the world have downloaded ngs.plot for hundreds of times. We have also created an online discussion group for users to ask questions and help one another. So far, there are 51 active members who have contributed to 69 topics. We also use this opportunity to collect opinions from users so that we can improve the program further.

### NGS data processing

The NGS data used in this manuscript were obtained from the Sequence Read Archive (SRA, http://www.ncbi.nlm.nih.gov/Traces/sra). The accession numbers and references of the datasets are listed in Table S1 [see Additional file 1]. ChIP-seq data were aligned to the reference genome by Bowtie [39]. Peak calling was accomplished by use of MACS [40] using default parameters. RNA-seq data were analyzed by the Tuxedo Suite [41]. The differential chromatin modification sites were detected by diffReps [42] using default parameters and the FDR cutoff was set as 0.1.

## Results and discussion

### Benchmarking the performance of ngs.plot

To benchmark different coverage extraction methods, we used a ChIP-seq dataset that we previously published [43]. H3K9me2 is a histone mark that displays dispersive enrichment patterns and is often associated with gene silencing [44]. The ChIP-seq samples were derived from a mouse brain region (nucleus accumbens) where two biological conditions were assessed: chronic morphine and chronic saline administration. For each condition, three biological replicates were analyzed. We merged and sorted the alignment BED files for the three biological replicates under saline conditions and used BEDTools [45] to create a large BAM file that contains nearly 250 million alignments. From this file, 10, 20, 40, 80, and 160 million alignments were randomly sampled to create a series of BAM files that increase in alignment size exponentially. Different methods were used to extract coverage vectors for the TSS ± 5 Kb regions of all protein coding genes (~20,000). A number of metrics such as run time, memory usage, and file size were measured for different alignment sizes. All tests were performed on a Linux workstation with two 2.4 GHz CPU cores and sufficient memory.

**Figure 3:**
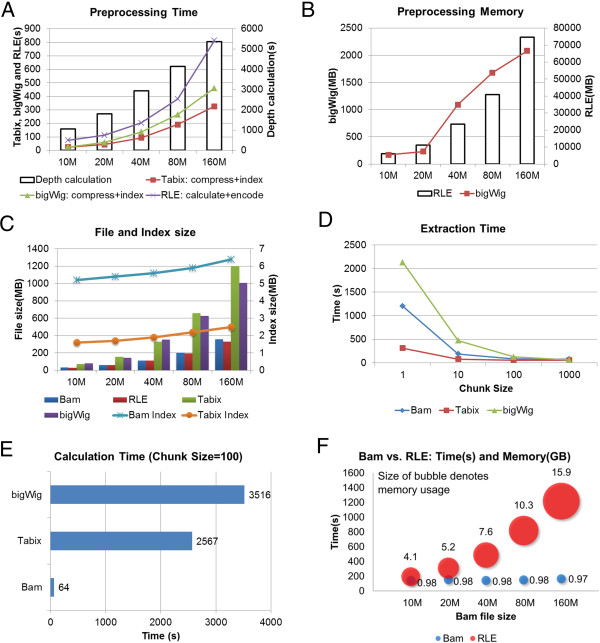
**Performance benchmark of different strategies. A**. Pre-processing time for different alignment sizes: Coverage calculation time for Tabix and bigWig is shown as vertical bars; Coverage compression and indexing combined time for Tabix and bigWig is shown as red square and green triangle trend lines, respectively; RLE calculates and encodes the coverage and the time is shown as a purple X-shape trend line. For the vertical bars, the scale is on the right y-axis. For the trend lines, the scale is on the left y-axis. **B**. Peak memory usage during pre-processing for different alignment sizes: RLE is shown as vertical bars whose scale is on the right y-axis; bigWig is shown as a red square trend line whose scale is on the left y-axis. **C**. File and index sizes for different alignment sizes: Bam, RLE, Tabix, and bigWig are shown as colour columns whose scale is on the left y-axis; Bam and Tabix index sizes are shown as trend lines whose scale is on the right y-axis. Please note that RLE, Tabix, and bigWig coverage files are all converted from BAM files, which incur extra storage. **D**. Alignment extraction time for all TSS ± 5 Kb regions on the mouse genome for different chunk sizes based on 10 million short reads. **E**. Coverage calculation time for all TSS ± 5 Kb regions on the mouse genome for chunk size of 100 based on 10 million short reads. **F**. Alignment extraction and coverage calculation combined time and peak memory usage for Bam and RLE for different alignment sizes. The size of the bubbles denotes memory usage and the vertical location of the bubble centers denotes time. Test is based on all TSS ± 5 Kb regions in the mouse genome.

At first, coverage needs to be pre-calculated for Tabix, bigwig, and RLE. This takes a long time to complete and the run time is strongly associated with the alignment size (Figure 3A). It takes samtools around 1,000 s to calculate the coverage for a 10 million read BAM file and more than 5,000 s for a 160 million read BAM file. RLE is much faster but involves a more rapid increase in time than samtools: it takes 80 s for a 10 million read BAM file and more than 800 s for a 160 million read BAM file. This is because RLE tries to load all alignments into memory and then performs calculations in a batch while samtools does the calculations by reading alignments in a stream. After coverage calculations, Tabix and bigWig also require the coverage files to be indexed. The indexing is more than 10 times faster than coverage calculation and shows strong association with the alignment file size (Figure 3A). Tabix is faster than bigWig: this is most likely because bigWig uses more than one index for different zoom levels [28].

Memory usage is a big problem for RLE. Even for the 10 million read BAM file, it uses 6 GB to finish the run, while for the 160 million read BAM file, it uses 75 GB (Figure 3B). In contrast, the memory footprint for Tabix indexing is very small: it uses ~50-60 MB for all BAM files. bigWig uses more memory for indexing than Tabix but is still reasonably small: at 160 million alignments, it uses 2 GB to finish the run (Figure 3B).

File size is another important metric. Both Tabix and bigWig create large coverage files that strongly associate with alignment file size (Figure 3C): at 160 million alignments, the Tabix coverage file is 1.2 GB while the bigWig coverage file is 1GB. As a comparison, RLE files are three times smaller: at 160 million alignments, the RLE file is 330 MB. Both Tabix and BAM have very small index file sizes. For BAM, the index remains around 6 MB for all alignment sizes while Tabix index is three times smaller. For bigWig, the index is an integral part of the format and its size is unknown to us.

By grouping regions into chunks we can save resource in index loading. This strategy worked well in our tests (Figure 3D). Based on a 10 million alignment file, it took the BAM method 1,200 s to load all TSS ± 5 Kb regions into memory for chunk size of 1. For a chunk size of 10, the time was reduced to less than 200 s – a six fold reduction. The time was further reduced to 88 s for a chunk size of 100. The other two methods – Tabix and bigWig – enjoyed similar degrees of time reduction by use of region grouping. It should be noted that Tabix used much less time than BAM at small chunk sizes. This is expected since the Tabix index is much smaller than the BAM index (Figure 3C). bigWig used the longest time among the three methods at small chunk sizes (1-100), suggesting its index is larger than the other two.

In our tests, Tabix was implemented with the Rsamtools [46] package and bigWig was implemented with the rtracklayer [47] package. Note that Tabix is a generic index program for text entries. After the texts are loaded into memory, they must be converted into binary representation of numerical numbers. The rtracklayer package, however, will unfortunately merge and sort the query regions before coverage vectors are retrieved. This means that the loaded coverage vectors are mixed and must be distinguished between the query regions for them to be useful for our purposes. All of the above operations require a significant amount of computational resources. At chunk size of 100, it took bigWig >3,500 s and Tabix >2,500 s to finish the operations (Figure 3E). In comparison, it took BAM only 64 s to calculate the coverage vectors on-the-fly. In the end, we abandoned support for Tabix and bigWig for this reason. A future goal of the field is to re-write the extraction functions in Rsamtools and rtracklayer extensively in order to optimize the retrieval time. Once that is done, we can add support for these two file formats.

Finally, we tested the entire process of coverage extraction and calculation for both BAM and RLE for different alignment sizes with regard to time and memory usage. The BAM method was tested with chunk size of 100 that is the default value for ngs.plot. BAM functioned superiorly compared to RLE on both metrics at all alignment sizes (Figure 3F). BAM’s run time only slightly increased from 143 s to 165 s for 10 and 160 million alignments; and its memory usage remained stable: less than 1 GB for all alignment sizes. In contrast, RLE used 4.1 GB RAM at 10 million alignments and increased to 15.9 GB RAM at 160 million alignments. RLE’s run time was also significant: at 160 million alignments, it took >1,200 s to finish – seven times longer than BAM.

In summary, the BAM strategy we chose in ngs.plot is a versatile, low profile approach that works robustly even with very large alignment files. This approach was introduced in ngs.plot v1.64 and has remained the approach of choice ever since.

### Analysis of Tet1 and 5hmC ChIP-seq data in the differentiation of P19.6 cells

An easy-to-use and flexible visualization method of NGS data is necessary for computational biologists to formulate and validate hypotheses quickly. To demonstrate the power of ngs.plot, we used ChIP-seq data [see Additional file 1: Table S1] to study the relationship between Tet1 (ten eleven translocation protein-1), a methylcytosine dioxygenase, and 5-hydroxymethycytosine (5hmC) in the differentiation of mouse embryonal carcinoma P19.6 cells. P19.6 cells can be differentiated into neurons or glia by exposure to retinoic acid (RA) [48], and are widely used in research on stem cell differentiation. Tet family proteins play important roles in the conversion of 5-methylcytosine (5mC) into 5hmC, 5-formylcytosine (5fC), and 5-carboxymethylcytosine (5caC) in DNA [49-51], and are important regulators in the maintenance and differentiation of embryonic stem cells (ESC) [52,53]. 5fC and 5caC are present low abundance in mammalian genomes [54] and are difficult to be detected by ChIP-seq. Therefore, we focus on 5hmC in this study. 5hmC is known to be enriched at TSSs, exons, CGIs, and enhancers [55-57]. The distributions of Tet proteins and 5hmC across the genome roughly overlap, while Tet1 and Tet3 prefer CpG enriched regions [50,58]. This preference is at least partially due to their CXXC domains [58].

**Figure 4:**
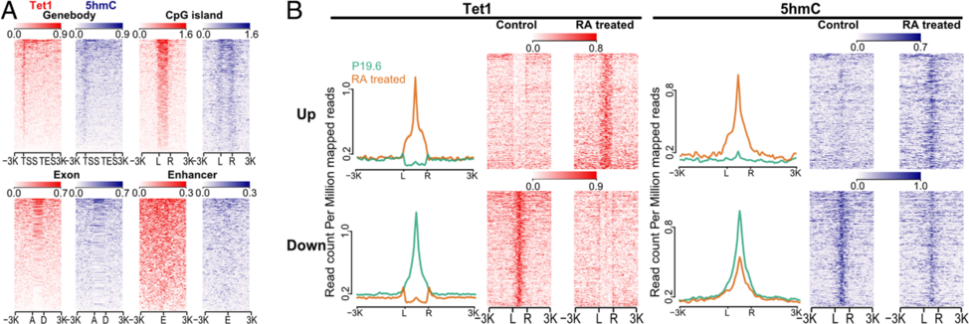
**Applying ngs.plot to the study of Tet1 in mESC P19.6 cells during differentiation.** All heatmaps are resized to match each other’s height for display purposes. **A**. Tet1 and 5hmC enrichment at different functional regions – CGIs at proximal promoters, canonical exons, and enhancers, including 3 Kb flanking regions. All regions are ranked by the “total” algorithm. “L” – 5’ left, “R” – 3’ right as defined by the gene that includes the CGI; “A” – 5’ acceptor, “D” – 3’ donor; “E” – enhancer center. **B**. Tet1 and 5hmC enrichment before and after RA treatment at Tet1’s differential sites defined by diffReps, filtered by active enhancers, including 3 Kb flanking regions. The differential sites are ranked by the “diff” algorithm. The up and down sites are plotted separately. Both average profiles and heatmaps are shown. “L” – genomic left, “R” – genomic right as lower coordinates are to the left of higher coordinates.

First, we used ngs.plot to investigate the enrichment profiles of Tet1 and 5hmC in P19.6 cells at different genomic regions, including genebodies, CGIs, exons, and enhancers (Figure 4A & Additional file 1: Figure S2). As the genebody plot (Figure 4A) shows, Tet1 is most enriched at TSSs but generally depleted at genebodies. CGI plots (Figure 4A & Additional file 1: Figure S2A) indicate that Tet1 is enriched at all kinds of CGIs at similar levels (~0.5-0.9 RPM) and demonstrates a clear drop of enrichment at flanking regions (±3 Kb). This suggests that the CXXC domain of the Tet1 protein highly prefers CpG abundant regions. In addition, Tet1 shows some enrichment at exons as well as enhancers (Figure 4A & Additional file 1: S2B) but the enrichment levels are weaker than that of CGIs, with enhancers being the weakest. As we expected, the enrichment patterns of 5hmC are highly similar to those of Tet1 (Figure 4A & Additional file 1: Figure S2), indicating concordance between the two marks. All of the above plots can be generated by ngs.plot with only one command for each. The user only needs to input into ngs.plot which regions and samples to examine and the size of the flanking regions.

5hmC plays an important role in stem cell differentiation, where its conversion from 5mC is mediated by Tet1 [49,51,55,59]. The activities of enhancers are known to be specific to differentiated cell types and are often marked by the dynamics of 5hmC [60]. Here we illustrate the role that Tet1 plays in the conversion of 5hmC by studying the differential sites of Tet1 between control and RA-induced P19.6 cells (Figure 4B & Additional file 1: Figure S2). diffReps is a powerful program to detect differential chromatin modification sites using ChIP-seq data [42]. We used diffReps to find 7,735 (Increased: 3,762, Decreased: 3,973) Tet1 differential sites in total. To restrict the analysis to enhancers, we used H3K27ac as a mark for active enhancers [33]. Peak calling using MACS was performed in both control and RA-induced P19.6 cells and the two peak lists were combined to obtain 135,280 H3K27ac enriched sites (excluding the TSS ± 3 Kb regions). The peak list was used to filter the Tet1 differential sites that are not in enhancer regions. After filtering, we obtained 507 increased and 1,875 decreased enhancer-specific Tet1 sites induced by RA, whose genomic coordinates are then converted into two separate BED files. ngs.plot was applied on each BED file to plot the enrichment of both Tet1 and 5hmC (Figure 4B). It can be seen clearly that the trends of 5hmC dynamics follow those of Tet1 dynamics, with an overall consistency ratio of 82% (Tet1 increased sites: 74%; decreased sites: 84%). Their log fold changes are also weakly correlated (Pearson’s r = 0.46, Spearman’s ρ = 0.32, both with P < 2.2E-16). This is a vivid example illustrating how different computational tools can be used to identify biologically meaningful genomic regions and then feed them into the ngs.plot program for visualization.

### Integrative analysis of poised and active promoters in ESC

Integrative analysis using genomic sequence information and multiple NGS samples is essential to investigate gene transcription and epigenomic regulation. ngs.plot’s ability to graph both ChIP-seq and RNA-seq samples allows a user to quickly establish correlations between different epigenomic marks and associated gene expression levels. Here, we demonstrate this feature of ngs.plot by use of multiple ChIP-seq samples, including several histone marks (H3K4me3, H3K27ac, and H3K27me3) and transcription factors (Suz12, Oct4, and Tet1), and an RNA-seq sample, from mouse ESCs (mESCs) [see Additional file 1: Table S1]. H3K4me3 is a promoter-enriched histone mark that is generally associated with transcriptional activation [33]. H3K27ac is an activation mark that locates at both promoters and enhancers [33]. The enrichment of both H3K4me3 and H3K27ac provides a signature of CpG-related promoters [61]. H3K27me3 is catalyzed by the Polycomb group proteins and is implicated in the silencing of genes [62]. The enrichment of both H3K4me3 and H3K27me3 marks the so-called “bivalent” domains that are prevalent in ESCs. They maintain the silencing or low expression of many genes in ESCs, which are poised for activation in differentiated cell types [33,63]. Suz12 is a subunit of the Polycomb repressive complex 2 (PRC2) – a transcriptional repressor that catalyzes H3K27me3 [64]. Oct4, also known as POU5F1, is a critical transcription factor in the self-renewal of ESCs [65].

**Figure 5:**
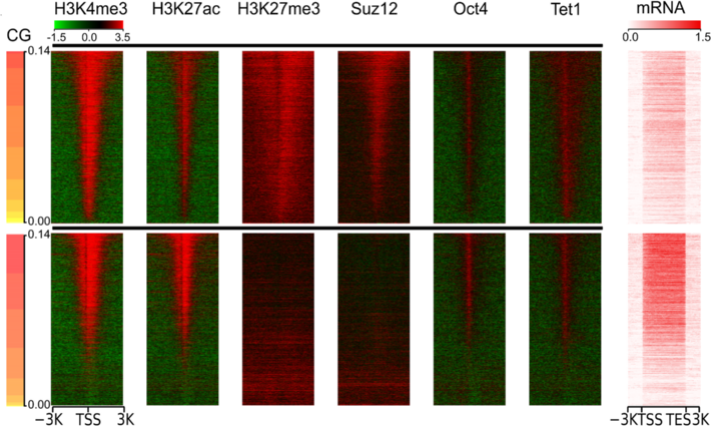
**Applying ngs.plot to the study of epigenomic regulation of PT and nPT promoters in mESCs.** The log2 enrichment ratios of several histone marks and transcription factors vs. DNA input at TSS ± 3 Kb regions. The TSSs are ranked by CGPs in descending order (using algorithm “none”). Gene expression levels are illustrated by RNA-seq enrichment in the same order (using RNA-seq mode), including 3 Kb flanking regions. The upper panel represents PT promoters and the lower panel represents nPT promoters. They are resized to have the same height.

We divided all promoters (TSS ± 3 Kb) of the coding regions of genes into two groups, namely, Polycomb-targeted (PT, n = 5,132) and non-Polycomb-targeted (nPT, n = 19,013), based on the presence or absence of H3K27me3 peaks. To reveal the relationship between genomic sequences and epigenomic regulation, we sorted all promoters within each group based on their CG di-nucleotide percentages (CGP) and entered the gene lists into ngs.plot’s configuration files. We ran ngs.plot with its ranking algorithm set to “none” so that it used the input order. We also used a DNA input sample to pair with each epigenomic mark so that ngs.plot’s bam-pair functionality plots log fold changes. The use of the input sample is to counteract various biases introduced in ChIP-seq experiments [66]. All of the ChIP-seq samples within each group were then plotted with one command by use of the configuration file (Figure 5). We also plotted the RNA-seq sample using the same gene list with another command using the “RNA-seq” mode (Figure 5).

Figure 5 shows that the PT group has lower gene expression levels than the nPT group, indicating that genes containing the H3K27me3 mark are suppressed. Conversely, the activation mark H3K27ac shows lower enrichment in the PT group. As previously reported [67], H3K27ac is mutually exclusive with H3K27me3. However, another activation mark, H3K4me3, appears to be enriched in both groups. H3K4me3’s enrichment in the PT group demonstrates the prevalent existence of the “bivalent” domain in mESCs. The heatmaps of Figure 5 also indicate that there are strong correlations between certain epigenomic marks as well as with gene expression. To quantitatively measure these correlations, we used the plotCorrGram.r script included in the ngs.plot package to calculate and visually demonstrate all pairwise correlations between the samples. All the correlation coefficients and p-values are presented in [Additional file 2]. The corrgram is presented in [Additional file 3]. As expected, H3K27me3 and Suz12 show very strong correlations in both groups (PT: r = 0.89, nPT: r = 0.94, both with P = 0). Interestingly, CGPs show a moderate correlation with gene expression in the nPT group (r = 0.69, ρ = 0.73, both with P = 0), but this correlation is significantly decreased in the PT group (r = 0.20, ρ = 0.24, both with P = 0). As we mentioned above, Tet1 has a preference for CG-rich regions due to its CXXC domain. A moderate correlation is observed between Tet1 and CGPs in the PT group (r = 0.53, ρ = 0.54, both with P = 0), while a weak correlation is observed in the nPT group (r = 0.30, ρ = 0.37, both with P = 0). Tet1 also shows a moderate correlation with Oct4 in both groups (PT: r = 0.59, 0.57, both with P = 0; nPT: r = 0.76, ρ = 0.71, both with P = 0). It has been reported that Tet1 can replace the role of Oct4 in inducible pluripotent stem cell (iPSC) reprogramming, a process that is implicated in the regulatory circuit of ESCs [68]. This example demonstrates the ngs.plot’s capability to quickly correlate multiple epigenomic marks with other genomic features and with gene expression and creates figures that are publication-ready. A user can use these figures to gain biological insights into their NGS data and even generate novel hypotheses.

### Examination of RNA-seq 3’ bias

**Figure 6:**
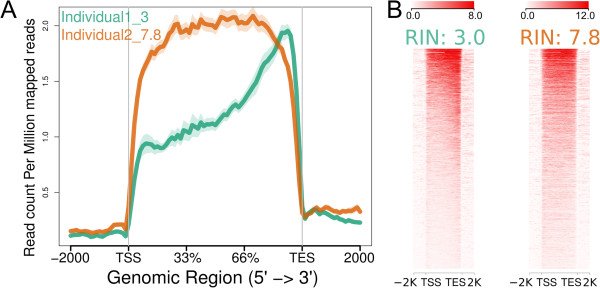
**RNA**-**seq plots of two human postmortem brain samples with different RIN values. A.** Average profiles. **B.** Heatmaps.

The RNA-seq mode of ngs.plot can perform exon splicing *in silico* and this functionality can be exploited during RNA-seq quality control. For instance, in studies of human postmortem brain tissue, a major problem is that the RNA samples are often severely and variably degraded, as measured by the RNA integrity number (RIN) [69]. An RNA sample with low RIN is often associated with strong 3’ bias, which can impair the ability to otherwise assess the sample’s mRNA quantity. To demonstrate this, we analyzed an in-house RNA-seq dataset (unpublished) from human postmortem brain tissue obtained from two individuals with schizophrenia: one sample has an acceptable RIN (=7.8) and the other sample has a very low RIN (=3). The figure (Figure 6) generated by ngs.plot shows that the sample with low RIN is clearly biased towards 3’ in comparison to the sample with high RIN. A plot like this provides a visual inspection of the read coverage of RNA-seq samples and can help an investigator derive useful information from suboptimal tissue, while guiding decisions regarding whether a sample should be discarded or not.

## Conclusion

High throughput assays that utilize NGS platforms have revolutionized biomedical research [1,2]. Biology is becoming more of a data-driven discipline than ever. The bottleneck is now in the processing and interpretation of the massive amount of data that are being generated [70,71]. We have developed ngs.plot – a quick data mining and visualization program for plotting NGS samples. ngs.plot is easy and simple to use but yet still very powerful. Its signature advantage is a built-in database of functional elements that are ready to use, which saves users considerable time in managing genomic coordinates on their own. These features help make ngs.plot a popular tool among bioinformatics researchers.

Over the past few years, we have seen many exciting developments in applying NGS technologies to epigenomics. Large international efforts such as the ENCODE project [11] and the NIH roadmap epigenomics project [72] have generated an enormous amount of data about the human and other mammalian genomes. The scale of such projects is unprecedented. These data have provided an invaluable resource of information concerning the functional elements that regulate genes and non-genic regions. Understanding how these functional elements are controlled by different protein regulators to yield numerous, diverse phenotypic outputs is essential to advance our knowledge of genome regulation and function. In this great adventure, ngs.plot represents a highly useful tool that helps fill the gap between data and information. Nevertheless, a lot of work is still needed to curate these data and to incorporate them into our database.

Another direction for future research is to make the ngs.plot program more interactive. As we incorporate tens of millions of additional functional elements into our database and perform more elaborate classifications, a command line interface will become too cumbersome to use. Therefore, a Google search like interface should be developed to help users find genomic regions of interest from our database, upon which the ngs.plot visualization engine can be used to display enrichment patterns and to perform related data mining tasks.

## Availability and requirements

### Project name

ngs.plot.

### Project home page

https://code.google.com/p/ngsplot/.

### Operating system

Platform independent.

### Programming language

R and Python.

### Other requirements

R package doMC; Bioconductor package BSgenome, Rsamtools and ShortRead.

### License

GNU GPL3.

### Any restrictions to use by non-academics

Contact Lisa Placanica (lisa.placanica@mssm.edu) or the technology transfer office of Mount Sinai.

## Abbreviations

NGS: Next-generation sequencing; TSS: Transcriptional start site; TES: Transcriptional end site; CGI: CpG island; DHS: DNase I hypersensitive sites; SEM: Standard error of mean; RLE: Run length encoding; GTF: Gene transfer format; GP: Gene prediction; 5hmC: 5-hydroxymethycytosine; RA: Retinoic acid; 5mC: 5-methylcytosine; 5fC: 5-formylcytosine; 5caC: 5-carboxymethylcytosine; ESC: Embryonic stem cells; PRC2: Polycomb repressive complex 2; CGP: CG di-nucleotide percentage; RIN: RNA integrity number.

## Competing interests

The authors declare that they have no competing interests.

## Authors’ contributions

LS designed and lead the development of the program, analysed the data and wrote the manuscript; NS contributed to the code, performed the computational experiments and drafted the manuscript; XL contributed the ngs.plot Galaxy plug-in; EN participated in the writing. All authors read and approved the final manuscript.

## Supplementary Material

Additional file 1 **Supplemental materials including exon classification algorithm, FigureS1-2, Table S1-2.** Click here for file

Additional file 2 **Correlation coefficients and p-values of all pairwise comparisons between the samples in Figure** 5. Click here for file

Additional file 3 **Corrgrams of histone marks, transcription factors, and gene expression using the same data as Figure** 5. Each region is represented by the row sum of the data matrix. The left panel represents PT promoters and the right panel represents nPT promoters. The upper triangle represents correlation coefficients: the sizes of pies represent the absolute values of the correlation coefficients; blue represents positive correlation; red represents negative correlation. The lower triangle represents scatter plots using ellipses. The red lines represent LOWESS fit to the scatter plots. Click here for file
