# Supplementary material for: ngs.plot: Quick mining and visualization of next-generation sequencing data by integrating genomic databases
Source: BMC Genomics. 2014 Apr 15;15:284. doi: 10.1186/1471-2164-15-284 (PMC4028082; doi:10.1186/1471-2164-15-284)
Supplement: Additional file 1 — Supplemental materials including exon classification algorithm, FigureS1-2, Table S1-2. [file 1471-2164-15-284-S1.DOC]

**Supplemental Materials**

**EXON CLASSIFICATION PROGRAM**

The exon classification program reads in a gene transfer format (GTF) file; parses the gene-transcript relationships and exon coordinates; then classifies the exons into seven categories; finally the program outputs the exon and genebody coordinates along with their classification. The genes’ information is stored in memory as a hash table whose data structure is illustrated in Fig. S1. The read, parse, and output of the gene table are trivial and not further explained here. The exon classification procedure is separated into two parts: algorithm 1 – ANNO_EXON is used to classify the exons of one transcript when it is compared with another transcript; algorithm 2 – ANNO_TABLE is used to classify exons of all genes and all transcripts.

The exons that are to be classified are named as “current” exons, while the exons that are compared against are named as “reference” exons. Line 3 of Algorithm 1 iterates through each exon of the current exons and performs classifications. Another iterator r is needed for reference exons and is initialized at line 2. After a reference exon is found to be on the right side of the current exon, the inner loop between 11 and 12 exists to perform classification on the current exon. The iterator r continuously increments through the loop between lines 3 and 38 because the next current exon can start from where it left off. When an exon is completely outside the body of another transcript, its type should stay the same as implemented on lines 13-16. Current exons may have already been classified during comparison with another transcript. This may lead to conflicts: for example, for transcripts A, B, and C, during the comparison of A and B, an exon is classified as “variant”, but during the comparison of A and C, the exon is classified as “altBoth”. Therefore, there must be a rule to resolve such conflicts. We set a priority of exon types as: variant > altBoth > other types. This is reflected on lines 9 and 20. A new classification may also lead to modifications to the existing classification. That is why a putative classification is first determined on lines 22-33. It is then compared with the existing classification to determine the new classification on lines 34-38.

Classifications for all genes and transcripts are rather straightforward. First, all exons for each transcript must be sorted by genomic coordinates on lines 2-3 of Algorithm 2. The algorithm then enumerates all possible pairwise comparisons between two transcripts of the same gene on lines 6-7. During a comparison, one transcript serves as reference and the other transcript is considered to be current. The order is then reversed to perform another classification. Lines 10-11 consider a degenerated case where only one transcript exists for the gene. Then the ANNO_EXON algorithm is simply used to annotate the 5’ and 3’ most exons to be promoter and polyA.

| **Algorithm 1 ANNO_EXON: Classify the exons of the current transcript by comparing them with the exons of the reference transcript.** | |
| --- | --- |
| **Input**:  ref_exons: exons of the reference transcript  cur_exons: exons of the current transcript  strand: strand (+/-) of the gene | |
| 1: | ref_exon_n <- number of ref_exons |
| 2: | r <- 0 |
| 3: | **foreach** cur_exon in cur_exons: |
| 4: | **if** cur_exon is 5' first: |
| 5: | cur_exon.class <- "promoter" |
| 6: | **else if** cur_exon is 3' last: |
| 7: | cur_exon.class <- "polyA" |
| 8: | **else**: |
| 9: | **if** cur_exon.class == "variant": |
| 10: | next |
| 11: | **while** r < ref_exon_n and cur_exon.end >= ref_exons[r].start: |
| 12: | r <- r + 1 |
| 13: | **if** r == 0: |
| 14: | next |
| 15: | **if** r == ref_exon_n and cur_exon.start > ref_exons[r-1].end: |
| 16: | next |
| 17: | **if** cur_exon not overlap with ref_exons[r-1]: |
| 18: | cur_exon.class <- "variant" |
| 19: | **else**: |
| 20: | **if** cur_exon.class == "altBoth": |
| 21: | next |
| 22: | putative_class <- None |
| 23: | **if** cur_exon overlaps with 5' most reference exon and they do not have the same 3'  boundary: |
| 24: | putative_class <- "altDonor" |
| 25: | **else** if cur_exon overlaps with 3' most reference exon and they do not have the same 5'  boundary: |
| 26: | putative_class <- "altAcceptor" |
| 27: | **else**: |
| 28: | **if** 5' boundaries are not the same for the two exons: |
| 29: | putative_class <- "altAcceptor" |
| 30: | **if** 3' boundaries are not the same for the two exons: |
| 31: | putative_class <- "altDonor" |
| 32: | **if** both 5' and 3' boundaries are not the same: |
| 33: | putative_class <- "altBoth" |
| 34: | **if** putative_class is defined: |
| 35: | **if** cur_exon.class == "altDonor" and putative_class == "altAcceptor" or vice versa: |
| 36: | cur_exon.class <- "altBoth" |
| 37: | **else**: |
| 38: | cur_exon.class <- putative_class |
| **Output**: annotated exons for the current transcript. | |

| **Algorithm 2 ANNO_TABLE: Annotate the whole gene table.** | |
| --- | --- |
| **Input**: unannotated gene table | |
| 1: | **foreach** gene in gene_table: |
| 2: | **foreach** transcript in gene: |
| 3: | sort the transcript's exons by genomic coordinates |
| 4: | trans_n <- total number of transcripts in the gene |
| 5: | **if** trans_n > 1: |
| 6: | **for** i in 0 to trans_n - 1: |
| 7: | **for** j in i to trans_n: |
| 8: | ANNO_EXON(transcript[i].exons, transcript[j].exons, gene.strand) |
| 9: | ANNO_EXON(transcript[j].exons, transcript[i].exons, gene.strand) |
| 10: | **else**: |
| 11: | ANNO_EXON(transcript[0].exons, transcript[0].exons, gene.strand) |
| **Output**: annotated gene table | |

**SUPPLEMENTAL FIGURES**


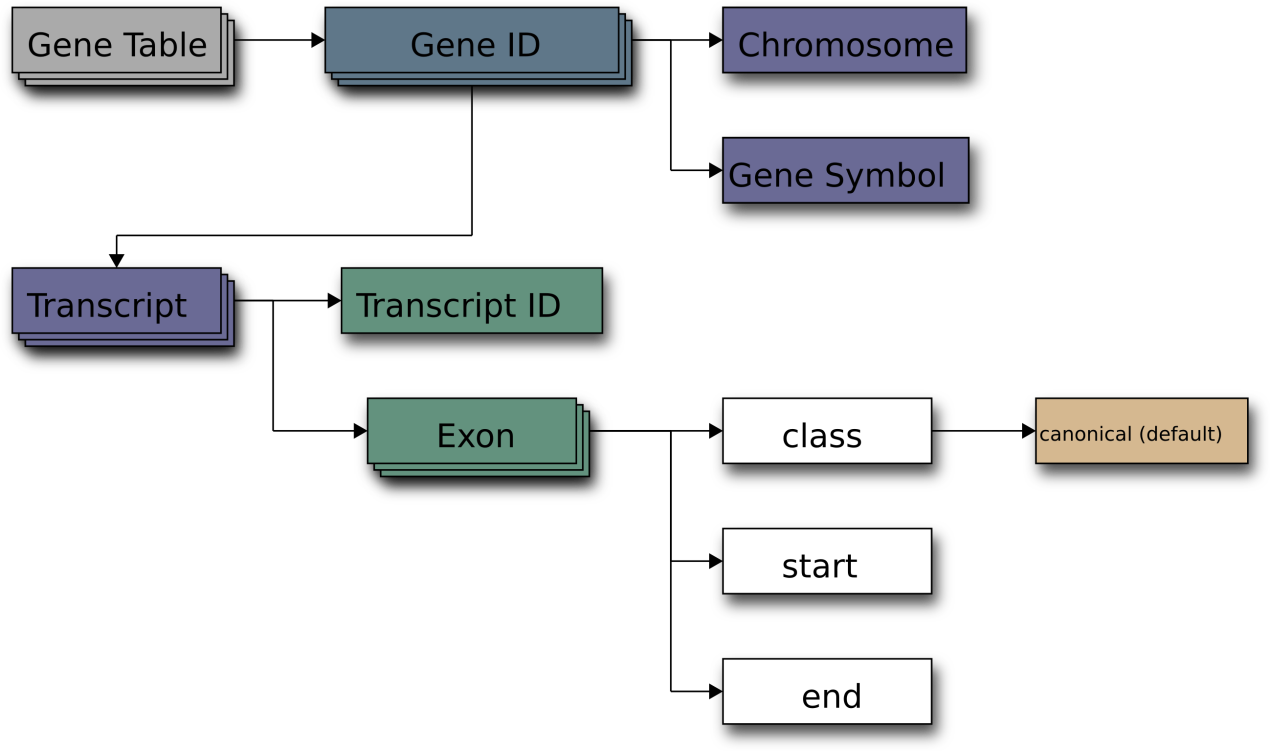


Figure S1. Data structure of the gene scheme table.





Figure S2. Tet1 and 5hmC enrichment patterns in control and RA-treated P19.6 cells. A. Tet1 average profiles at different types of CGIs. B. Tet1 average profiles at different types of exons. C. Tet1 and 5hmC enrichment before and after RA treatment at Tet1’s differential sites defined by diffReps, and filtered by active promoters (using H3K27ac), including 3 Kb flanking regions. The differential sites are ranked by the “diff” algorithm. The up (n=165) and down (n=323) sites are plotted separately. The up sites have a consistency score of 18% and the down sites have a consistency score of 93%. The overall consistency score is 68% with r=0.31 (P=5.1E-12) and ρ=0.25 (P=4.4E-16). Both average profiles and heatmaps are shown. The heatmaps are resized to have the same height. “L” – genomic left, “R” – genomic right as lower coordinates are to the left of higher coordinates.

**SUPPLEMENTAL TABLES**

Table S1. Accession numbers and references of the datasets used in this manuscript.

| **Type** | **Accession ID** | **Reference** |
| --- | --- | --- |
| **mESCs:** | | |
| H3K27ac | GSM594579, GSM594578 |  |
| H3K27me3 | GSM307619 |  |
| H3K4me3 | GSM723017 |  |
| Suz12 | GSM288360 |  |
| Tet1 | GSM611194 |  |
| Oct4 | GSM1082340 |  |
| mRNA | GSM723776 |  |
| **P19.6 cells** | | |
| Tet1 | GSM941680 |  |
| 5hmC | GSM941664 |  |
| Input | GSM821509 |  |
| **RA-treated P19.6 cells:** | | |
| Tet1 | GSM941681 |  |
| 5hmC | GSM941665 |  |
| Input | GSM821510 |  |

Table S2. URLs of the public databases used by the genome crawler.

| Database | URL |
| --- | --- |
| ENSEMBL | <ftp://ftp.ensembl.org/pub>; <ftp://ftp.ensemblgenomes.org/pub> |
| UCSC | [http://genome.ucsc.edu](http://genome.ucsc.edu/) |
| ENCODE | <http://genome.ucsc.edu/ENCODE>; <http://chromosome.sdsc.edu/mouse/download.html> |

**REFERENCES**

1. Creyghton, M.P., Cheng, A.W., Welstead, G.G., Kooistra, T., Carey, B.W., Steine, E.J., Hanna, J., Lodato, M.A., Frampton, G.M., Sharp, P.A. *et al.* (2010) Histone H3K27ac separates active from poised enhancers and predicts developmental state. *Proc Natl Acad Sci U S A*, **107**, 21931-21936.

2. Mikkelsen, T.S., Ku, M., Jaffe, D.B., Issac, B., Lieberman, E., Giannoukos, G., Alvarez, P., Brockman, W., Kim, T.K., Koche, R.P. *et al.* (2007) Genome-wide maps of chromatin state in pluripotent and lineage-committed cells. *Nature*, **448**, 553-560.

3. Shen, Y., Yue, F., McCleary, D.F., Ye, Z., Edsall, L., Kuan, S., Wagner, U., Dixon, J., Lee, L., Lobanenkov, V.V. *et al.* (2012) A map of the cis-regulatory sequences in the mouse genome. *Nature*, **488**, 116-120.

4. Chen, X., Xu, H., Yuan, P., Fang, F., Huss, M., Vega, V.B., Wong, E., Orlov, Y.L., Zhang, W., Jiang, J. *et al.* (2008) Integration of external signaling pathways with the core transcriptional network in embryonic stem cells. *Cell*, **133**, 1106-1117.

5. Williams, K., Christensen, J., Pedersen, M.T., Johansen, J.V., Cloos, P.A., Rappsilber, J. and Helin, K. (2011) TET1 and hydroxymethylcytosine in transcription and DNA methylation fidelity. *Nature*, **473**, 343-348.

6. Whyte, W.A., Orlando, D.A., Hnisz, D., Abraham, B.J., Lin, C.Y., Kagey, M.H., Rahl, P.B., Lee, T.I. and Young, R.A. (2013) Master transcription factors and mediator establish super-enhancers at key cell identity genes. *Cell*, **153**, 307-319.

7. Sérandour, A.A., Avner, S., Oger, F., Bizot, M., Percevault, F., Lucchetti-Miganeh, C., Palierne, G., Gheeraert, C., Barloy-Hubler, F., Péron, C.L. *et al.* (2012) Dynamic hydroxymethylation of deoxyribonucleic acid marks differentiation-associated enhancers. *Nucleic acids research*, **40**, 8255-8265.
