# Supplementary figures and images for: ngs.plot: Quick mining and visualization of next-generation sequencing data by integrating genomic databases
Source: BMC Genomics. 2014 Apr 15;15:284. doi: 10.1186/1471-2164-15-284 (PMC4028082; doi:10.1186/1471-2164-15-284)

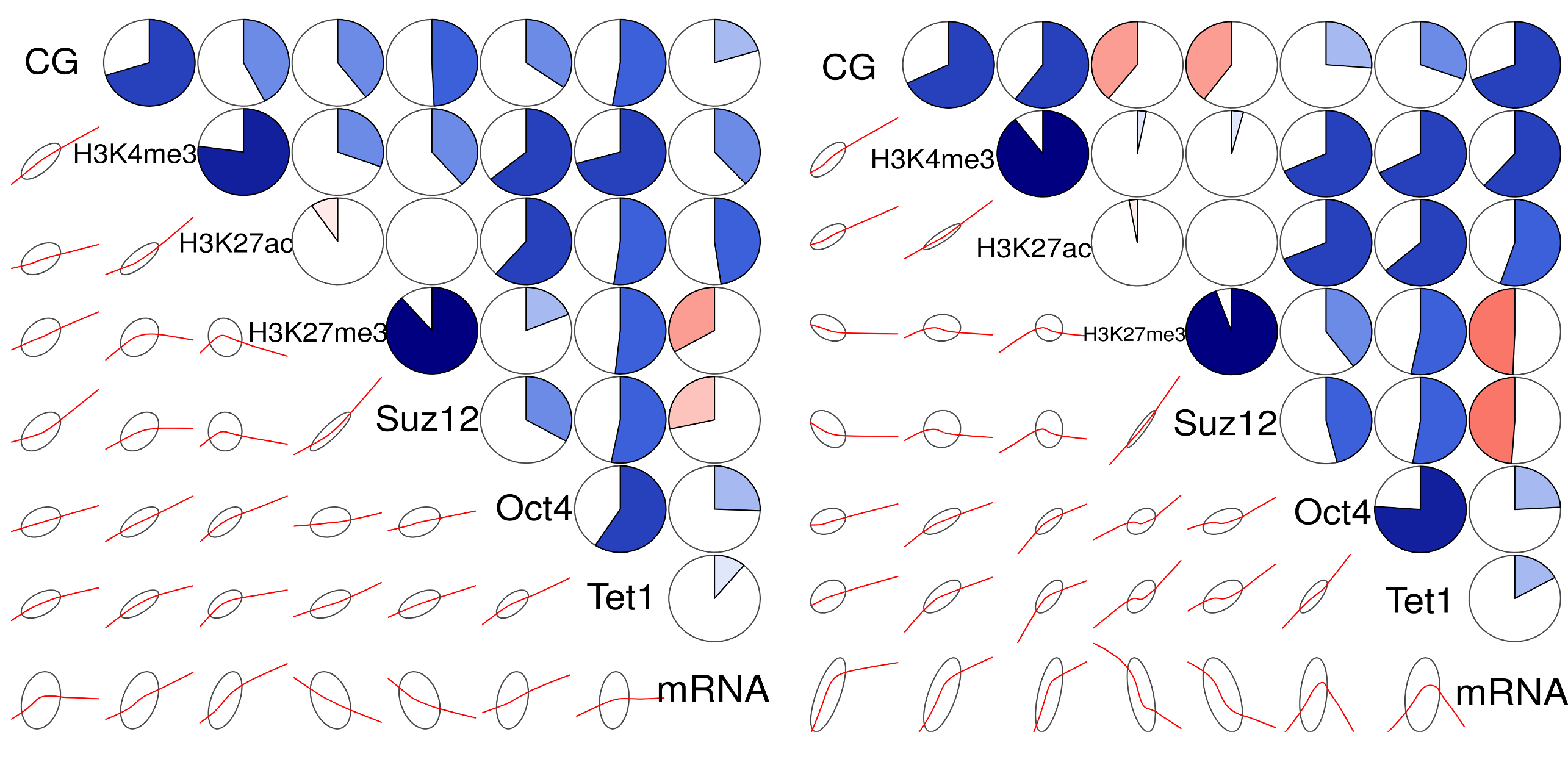

Supplement: Additional file 3 — Corrgrams of histone marks, transcription factors, and gene expression using the same data as Figure 5. Each region is represented by the row sum of the data matrix. The left panel represents PT promoters and the right panel represents nPT promoters. The upper triangle represents correlation coefficients: the sizes of pies represent the absolute values of the correlation coefficients; blue represents positive correlation; red represents negative correlation. The lower triangle represents scatter plots using ellipses. The red lines represent LOWESS fit to the scatter plots. [file 1471-2164-15-284-S3.PNG]
